# Zonal Chemical Signal Pathways Mediating Floral Induction in Apple

**DOI:** 10.3390/metabo14050251

**Published:** 2024-04-25

**Authors:** Priyanka Reddy, Tim Plozza, Alessio Scalisi, Vilnis Ezernieks, Ian Goodwin, Simone Rochfort

**Affiliations:** 1 Agriculture Victoria, AgriBio, Centre for AgriBioscience, Bundoora, VIC 3083, Australia; 2 School of Applied Systems Biology, La Trobe University, Bundoora, VIC 3083, Australia; 3 Tatura SmartFarm, Agriculture Victoria, Tatura, VIC 3616, Australiaian.goodwin@agriculture.vic.gov.au (I.G.); 4 Centre for Agricultural Innovation, University of Melbourne, Parkville, VIC 3010, Australia

**Keywords:** *Malus domestica* Borkh, return bloom, metabolomics, leaders, apple bud, biennial bearing, cytokinins, ABA, ‘Ruby Matilda’

## Abstract

Phytohormones that trigger or repress flower meristem development in apple buds are thought to be locally emitted from adjacent plant tissues, including leaves and fruitlets. The presence of fruitlets is known to inhibit adjacent buds from forming flowers and thus fruits. The resulting absence of fruitlets the following season restores flower-promoting signalling to the new buds. The cycle can lead to a biennial bearing behaviour of alternating crop loads in a branch or tree. The hormonal stimuli that elicit flowering is typically referred to as the floral induction (FI) phase in bud meristem development. To determine the metabolic pathways activated in FI, young trees of the cultivar ‘Ruby Matilda’ were subjected to zonal crop load treatments imposed to two leaders of bi-axis trees in the 2020/2021 season. Buds were collected over the expected FI phase, which is within 60 DAFB. Metabolomics profiling was undertaken to determine the differentially expressed pathways and key signalling molecules associated with FI in the leader and at tree level. Pronounced metabolic differences were observed in trees and leaders with high return bloom with significant increases in compounds belonging to the cytokinin, abscisic acid (ABA), phenylpropanoid and flavanol chemical classes. The presence of cytokinins, namely adenosine, inosine and related derivatives, as well as ABA phytohormones, provides further insight into the chemical intervention opportunities for future crop load management strategies via plant growth regulators.

## 1. Introduction

All newly formed apple buds contain leaf or shoot primordia and floral transformation of these buds only begins around the time when fruitlets emerge during late spring. Flower organ development stages of the bud occur through the season, including floral induction (FI), floral initiation and floral differentiation. Maturation of the bud is achieved during winter before bud-break occurs the following spring, when the phenological cycle begins again.

FI is when chemical signals are received by vegetative buds to induce floral meristem development. The exact time point of the FI phase is unclear; however, it is hypothesised to take place 60–70 days after full bloom (DAFB) in many apple cultivars, approximately 2 weeks prior to floral initiation [1,2] when the first structural changes occur in the bud. Floral initiation is visible microscopically via histological sectioning. It is about 12 weeks after bloom when the morphological changes are visible, marked by the appearance of the dome-shaped apex in the bud known as the flower differentiation phase [3]. Flower buds are most visibly distinct from vegetative buds during winter when they enter maturation.

Previous reports have indicated that it is likely that chemicals emitted by fruitlet seeds are responsible for floral repression, as adjacent buds are likely to remain vegetative and develop into leaves or shoots [4,5,6]. The removal of fruitlets via thinning practices during late spring, prior to the FI phase, have been shown to result in a more consistent return bloom, thereby an even crop load for the following season. Thinning practices that occur post-FI mostly improve the quality of the current season’s crop load, indicating that the fruitlets have a significant role to play in the chemical signalling mechanism for flower organ development. A high number of fruitlets on a tree during the FI phase results in suppression of return bloom that in turn generates a low crop load for the following season. The tree would then enter an alternating cropping cycle, known as biennial bearing. Trees that bear high crop loads are often described as ‘ON’ trees and those with relatively lower yields are described as ‘OFF’ trees [1,2].

While there are many studies that have investigated the physiological pathways of biennial bearing, the triggers and source of FI and repression are still largely unknown [1,2,4,7,8]. The negative correlation between fruitlet development and flower bud formation in strongly biennial bearing cultivars is the most studied cause of flower set variability. Fruitlet development over the flower bud initiation rate was believed to be one of nutritional competition due to inhibition of flower formation with the concurrent development of fruit [9]. The hypothesis was challenged by Chan and Cain [10], with experiments demonstrating that fruit number in parthenocarpic cultivars with seedless fruit had little effect on flower bud formation compared to seeded fruit, which had inhibitory effects unless removed after bloom. Thus, the study demonstrated that the rich and diverse phytohormones present in seed may be a factor contributing to biennial bearing [2]. Milyaev et al. [2] investigated the hypothesis and suggested that compounds involved in floral repression should be present in fruit flesh and in relatively higher abundance in trees with high floral repression (i.e., ‘ON’ trees) compared to trees that would largely induce flowering (i.e., ‘OFF’ trees). However, there were no correlations between compounds present in seed and those in buds, and thus, it remains in question whether phytohormones present in seed are in fact diffused into buds.

In vitro studies showed that flowering in the model plant *Plumbago indica* was induced by sugars and a mix of certain plant hormones, including cytokinins, adenine and low levels of auxin and inhibited by the vitamin riboflavin, the gibberellin class of plant hormones and amino acids such as glutamine and asparagine [11,12]. Genetics [4] and gene expression studies including transcripts [1,13] and multi-omics investigations [1] associated with biennial bearing suggested the role of sugar metabolism (carbohydrates) and hormone-related genes as likely candidates involved in biennial bearing. The multi-omics studies also indicated that thiamine, chlorogenic acid and an adenine derivative are involved in flower bud development in apple [1]. Increased levels of flavonoids such as kaempferol derivatives were also identified in low-crop-load trees.

The exogenous application of metabolites can engineer new pathways within a plant that mimic a response to stress conditions [14,15,16], further validating the involvement of plant hormones as a trigger for FI. The use of the endogenous or synthetic hormonal growth regulators to crop load in pome fruit is widespread. The synthetic cytokinin 6-benzyladenine (BA) is an effective post-bloom thinner for apple, as it reduces crop load, improves fruit quality and increases the return bloom for the following season [17]. Although variable results were observed with abscisic acid (ABA) as a thinning agent, it is naturally produced in plants as a growth regulator by ethylene biosynthesis and up-regulation of corresponding genes and plays a significant role in responding to abiotic and biotic stress [18,19]. In combination with BA, salicylic acid, a natural phytohormone, likely associated with FI, improved the return bloom in the apple cultivar ‘Northern Spy’ [20]. Reddy et al. [21] explains that buds in the FI phase in the strongly biennial cultivar ‘Nicoter’ produce salicylates, and in the less biennial bearing cultivar ‘Rosy Glow’, there is additional evidence of cytokinin involvement via the His-Asp pathway.

Chemical signalling is further substantiated by the formation of zones within trees that show biennial behaviour independent to the rest of the tree. Zones were generally branches or areas (i.e., tree tops) that started with high flower numbers but became biennial, while the rest of the tree showed relatively constant flower numbers. The fact that FI in these zones is different from the rest of the tree indicates short-range signalling [22].

Expression of phytohormones and carbohydrates can be specific to rootstock [5] and cultivar [1,21]. Chlorogenic acid—a key chemical associated with FI in apple—can differ in abundance and structural diversity in regular bearers compared to biennial bearers. Similarly, the same studies showed that tryptophan and metabolism of tryptophan, an upstream precursor for auxin and indoleamine production, varied in cultivar and treatments. Together, this suggests that the metabolic pathways activated in response to crop load levels are distinct in different cultivars in apple.

It would be beneficial for the apple industry to identify zonal signalling metabolites that could be artificially manipulated in trees or tree sections to balance flowering within the whole tree. To determine the metabolic pathways involved in FI in a less susceptible biennial bearing cultivar with a two-leader system, an untargeted metabolomics analysis of buds was performed for this study. Buds were collected from young ‘Ruby Matilda’ apple trees 60 DAFB, two weeks prior to the floral initiation period. Metabolites were analysed from bud samples harvested from leaders with a range of crop load treatments using high-resolution mass spectrometry. Here we describe the identification of key metabolites associated with FI using MS2 fragmentation and structure elucidation.

## 2. Materials and Methods

### 2.1. Experimental Site and Crop Load Treatments

The experiment was conducted in a commercial ‘Ruby Matilda’ apple (marketed as Pink Lady^®^) orchard (Plunkett Orchards, Ardmona, VIC, Australia). A randomised block design with 3 blocks consisting of 10 trees each was used. Trees were trained to bi-axis with two leaders labelled ‘primary’ (L1) and ‘secondary’ (L2) leader. Flower clusters on each leader were manually counted at full bloom (70–80% open flowers).

The return bloom dataset was categorised for ‘Ruby Matilda’ treatments denoted as RT^HIGH^ (12.6–20.0 flower no. cm^−2^ leader cross-sectional area (LCSA); 4.0–10.1 fruit no. cm^−2^ LCSA; *n* = 12), RL^HIGH^ (15.4–21.6 flower no. cm^−2^ LCSA of leader; 3.3–17.2 fruit no. cm^−2^ LCSA of leader; *n* = 12), RT^LOW^ (2.93–4.48 flower no. cm^−2^ LCSA; 2.24–6.46 fruit no. cm^−2^ LCSA; *n* = 12), and RL^LOW^ (2.09–3.95 flower/cm^2^ LCSA of leader; 5.0–7.5 fruit no. cm^−2^ LCSA of leader; *n* = 12).

The standard commercial thinning practice of 120 fruit/leader was used as reference crop load treatment. In addition, the other crop load treatments imposed to L1 leaders were 20, 70, 170 and 220 fruit/leader. Crop load treatments were imposed within 5–6 weeks of full bloom to alter the following year’s return bloom. L2 leaders were subjected to two crop load levels—‘low’ (<50 fruit/leader) and high (>200 fruit/leader).

### 2.2. Apple Bud Preparation and Metabolomic Extraction

Buds were harvested post-thinning from apple trees in late spring and early summer of the 2020/2021 growing season. Collection of three buds per leader occurred 60 DAFB. Bud selection and preparation is as previously described [21]. Briefly, buds were harvested from spurs and placed on ice before the scales were removed. Samples were stored at −80 °C prior to metabolomics analysis.

The metabolite extraction protocol was performed as reported previously by Reddy et al. [21]. Briefly, apple buds were lyophilised and ground using yttria zirconia beads on 24-well cryo-blocks on a Geno/Grinder 2010 [21]. The samples were extracted with 80% methanol/water (*v*/*v*), with extraction volumes adjusted proportionally to the weight of the lyophilised bud, and centrifuged. A 200 μL aliquot of the supernatant was transferred into an HPLC tube and stored at −20 °C until ready for LCMS analysis [21].

### 2.3. LCMS Methods for Untargeted and Targeted Analysis

The untargeted metabolite LCMS and LCMSMS profiling method is described in full in Reddy et al. [21]. Briefly, a Vanquish ultra-high performance liquid chromatography (UHPLC) system (Thermo Fisher Scientific, Bremen, Germany) coupled with a QExactive (QE) Plus mass spectrometer (Thermo, Bremen, Germany) with electrospray (ESI) probe, operating in both positive and negative modes, was used. Samples were randomised, and blanks (80% methanol) injected every five samples. A pooled biological quality control (PBQC) was run every 10 samples. For MS^2^, data were acquired in full-scan MS/data-dependent MS^2^ (ddMS^2^) mode on positive and negative ionisation modes on selected samples. Prior to data acquisition, the system was calibrated with Pierce LTQ Velos ESI Positive and Negative Ion Calibration Solution (Thermo Fisher Scientific). Mass spectrometry data were acquired using Thermo Xcalibur V. 2.1 (Thermo Fisher Scientific Inc., Waltham, MA, USA). Nitrogen was used as the sheath, auxiliary and sweep gases at flow rates of 28, 15 and 4 L/min, respectively. Spray voltage was set at 4000 V (positive and negative).

A Thermo Fisher Scientific Hypersil Gold 1.9 μm, 100 mm × 2.1 mm column was used with a mobile phase consisting of 0.1% formic acid in H_2_O (A) and 0.1% formic acid in acetonitrile (B). The mobile phase gradient is described in Reddy et al. [21]. In short, a flow rate of 0.3 mL/min of 2% B, increasing to 100% B, followed by 2% equilibration over 20 min was used.

### 2.4. Data Processing and Statistical Analyses

The data files obtained following LCMS analyses were processed in the Refiner MS module of Genedata Expressionist^®^ 12.0 with parameters as previously described [21].

Statistical analyses were performed using the Analyst module of Genedata Expressionist^®^ 12.0. Principal component analyses (PCAs) were performed to identify differences in leader and tree response. Overlay of the PBQC and samples allowed for the validation of the high-quality dataset by ensuring RT variation, mass error and sensitivity changes throughout. Identification of metabolites was performed by searching experimental MS^1^ data through the following databases: Plant Metabolic Network (PMN) (https://plantcyc.org) (accessed on 25 January 2023); Human Metabolome DataBase (HMDB) (http://hmdb.ca) (accessed on 25 January 2022); ChemSpider (http://chemspider.com) (accessed on 25 February 2023); and Lipid Maps^®^ (http://www.lipidmaps.org) (accessed on 20 June 2022). MS^2^ data were searched on MzCloud (https://www.mzcloud.org) (accessed on 25 March 2023).

Previously described linear models and OPLSDA models was applied to each dataset using MetaboAnalyst 3.053 [23] with missing value imputation on individual leaders of the ‘Ruby Matilda’ cultivar, revealing significant metabolites, as indicated by a *p*-value of <0.05 [21].

## 3. Results

To investigate zonal chemical signals associated with biennial bearing in apples, crop load treatments were applied to individual leaders in the cultivar ‘Ruby Matilda’ and apple buds collected 60 days post-crop load treatments and analysed using ultra-high performance liquid chromatography–high resolution mass spectrometry (UHPLC-HRMS).

Principal component analysis (PCA) plots (Figure S1) on UHPLC-HRMS metabolite dataset indicated reproducible pooled biological quality control (PBQC) data. Initially, the dataset revealed no clear separation across treatments; however, high- and low-return bloom data for individual leaders and at tree level indicated some separation between the two treatment types (Figure S2).

An orthogonal projection to latent structure discriminant analysis (OPLS-DA) model was utilised on only high- and low-return bloom data for positive and negative ionisation mode whole datasets for individual leaders and tree level. The OPLS-DA score plot revealed separation between treatment groups in both the positive and negative mode datasets.

**Figure 1:**
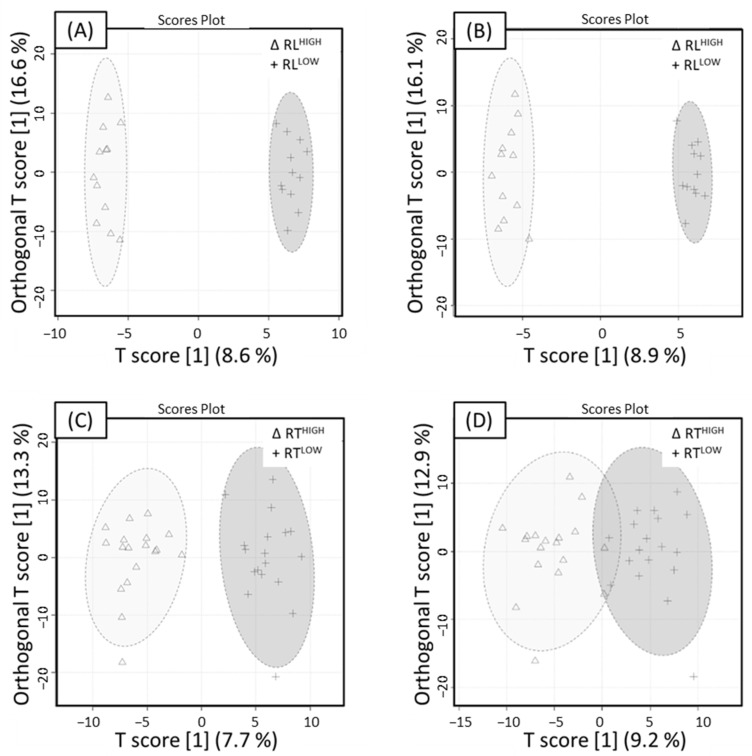
Orthogonal partial least squares discriminant analysis (OPLS-DA) of RL^LOW^ and RL^HIGH^ extracts acquired in UHPLC-HRMS. RL^LOW^ (+) and RL^HIGH^ (Δ) OPLS-DA score plot for (**A**) ESI+ mode with an associated 95% confidence ellipses (Q^2^ = 0.40, R^2^Y = 0.59) and (**B**) ESI− mode with an associated 95% confidence ellipses (Q^2^ = 0.39, R^2^Y = 0.58). RT^LOW^ (+) and RT^HIGH^ (Δ) OPLS-DA score plot for (**C**) ESI+ mode with an associated 95% confidence ellipses (Q^2^ = 0.43, R^2^Y = 0.69) (**D**) ESI− mode with an associated 95% confidence ellipses (Q^2^ = 0.41, R^2^Y = 0.68). All models were significant, indicated by 100 different model permutations for (**A**) (*p* < 0.01, Q^2^ = 0.61 and R^2^Y = 0.99), (**B**) (*p* < 0.01, Q^2^ = 0.59 and R^2^Y = 0.99), (**C**) (*p* < 0.01, Q^2^ = 0.62 and R^2^Y = 0.92) and (**D**) (*p* < 0.01, Q^2^ = 0.54 and R^2^Y = 0.79).

To investigate short-range signalling pathways in ‘Ruby Matilda’, the dataset was evaluated using metadata from individual leaders and at the tree level. For individual leaders, model performance of the positive mode data was well-described with moderate predictive performance with RL^LOW^ and RL^HIGH^ (Q^2^ = 0.40, R^2^Y = 0.59), as shown in Figure 1A. Using 100 different model permutations (Q^2^ = 0.62, *p* < 0.01 and R^2^Y = 0.99) indicated the model was significant. Negative mode for RL^LOW^ and RL^HIGH^, shown in Figure 1B, performed moderately (Q^2^ = 0.39, R^2^Y = 0.58). Using 100 different model permutations (*p*-value < 0.01, Q^2^ = 0.59 and R^2^Y = 0.99) indicated the model was significant. Variable importance in projection (VIP) was used to identify compounds mainly responsible for the separation in OPLS-DA models. Metabolites that resulted in a VIP > 2.0 were used as a threshold for variable selection. A total of 19 significant metabolites in the positive mode and 15 metabolites in the negative mode were identified.

Whole tree signalling pathways in ‘Ruby Matilda’ were also investigated. Model performance for positive mode was well-described in RT^LOW^ and RT^HIGH^ (Q^2^ = 0.43, R^2^Y = 0.69) data, as shown in Figure 1C. Using 100 different model permutations (*p* < 0.01, Q^2^ = 0.62 and R^2^Y = 0.92), the model was found to be significant. Good predictive performance (Q^2^ = 0.41, R^2^Y = 0.68) of negative mode data for RT^LOW^ and RT^HIGH^ treatments is shown in Figure 1D. Using 100 different model permutations (*p*-value < 0.01, Q^2^ = 0.54 and R^2^Y = 0.79), the model was found to be significant. Variable selection of positive and negative mode data revealed a total of 25 and 23 metabolites, respectively, using a threshold of VIP > 2.0.

A linear model (y (metabolite response) ~ flower cluster numbers per leader (LCSA)) was applied to the individual leaders in ‘Ruby Matilda’ for whole datasets in positive and negative mode. The flower cluster data were treated as an independent continuous variable for each cultivar. Metabolites in the apple spur buds were mostly elevated in RT^HIGH^ treatments compared to RT^LOW^ (Table 1 and Table 2), consistent with previous studies [21].

Library matching of MS or MSn fragmentation of parent ion confirmed the identification of differentially expressed metabolites for ‘Ruby Matilda’ in the whole tree (Table 1 and Table 2) and in leaders (Table 3 and Table 4) representing zonal signalling. Level 3 identification or above is required for putative identification of compounds in accordance with the Metabolomics Standards Initiative and Schrimpe-Rutledge et al. [21,22]. Most metabolites in Table 1, Table 2, Table 3 and Table 4 had level 2 identification, i.e., compounds that have matching fragmentation pattern with metabolite MS/MS libraries. Level 3 identification is ascribed when tentative structures are elucidated from database searches and the compound likely belongs to a particular chemical class. Level 4 identification arises when a molecular formula is only derived from the *m*/*z* molecular feature, rendering many structural possibilities. A level 5 identification represents a deconvoluted *m*/*z* molecular feature.

Many phenylpropanoid derivatives were assigned level 3 identification, as characteristic MS/MS ions, including *m*/*z* 265, 163 and 145, indicated the presence of structural analogues.

**Figure 2:**
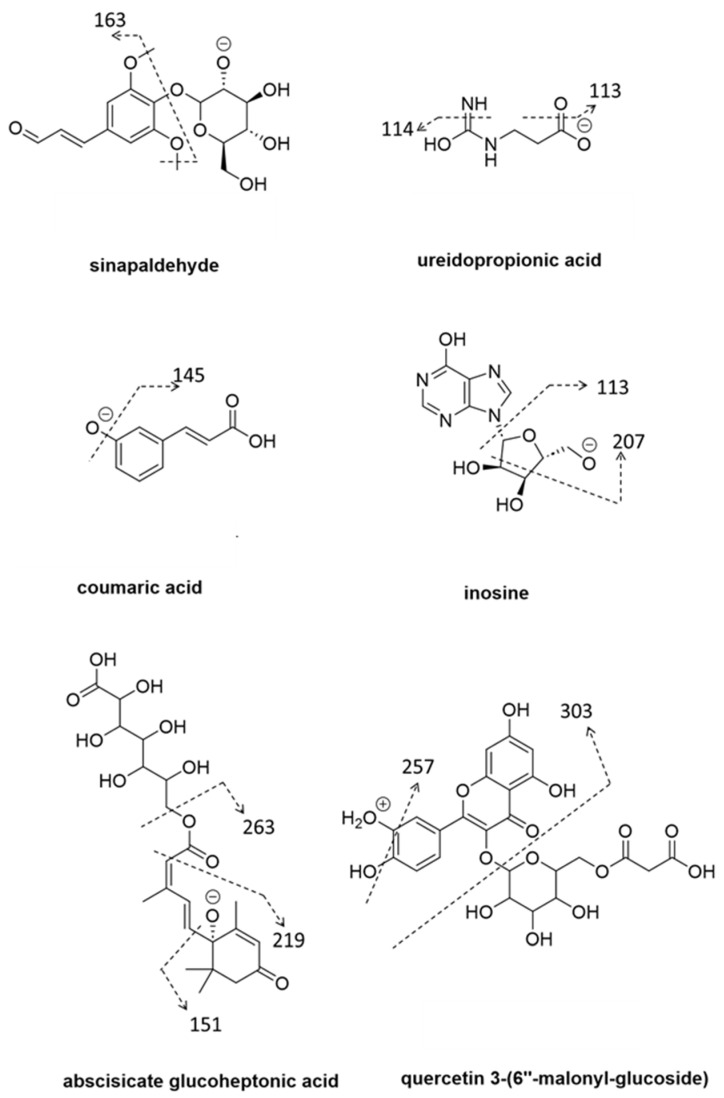
Selected examples of compounds and key fragments representing chemical classes associated with development of floral meristem in ‘Ruby Matilda’. These include phenylpropanoids: sinapaldehyde, coumaric acid; cytokinins: inosine; ABA: abscisate glucoheptonic acid; flavanol: quercetin.

The majority of metabolites were elevated in buds that were harvested from trees with high return bloom. Figure 2 shows some selected examples of compounds representing phenylpropanoid, cytokinin and flavanol chemical groups, associated with FI.

## 4. Discussion

Key compounds associated with FI, representing chemical signals within leaders and in the whole tree, were determined using positive and negative LCMS metabolic profiling on apple buds collected within the FI time period (60 DAFB). The overall high levels of cytokinins, flavanols, hydroxycinnamic acid and cinnamate derivatives in trees with high return bloom indicated disruption in the phenylpropanoid and cytokinin biosynthesis pathway. These results were consistent with previous studies on cultivars ‘Nicoter’ and ‘Rosy Glow’ [21] as well as ‘Fuji’ and ‘Gala’ [1]. The metabolic pathways associated with FI can vary between cultivars and root stocks depending on their susceptibility to biennial bearing and crop load treatments [1,7,21,24]. Previous studies on the cultivar ‘Rosy Glow’—mildly susceptible to biennial bearing—indicated cytokinin involvement due to the presence of the downstream signalling peptide His-Asp in trees with high FI (‘OFF’ trees), although the related pathway intermediates were not identified.

In this study, ‘Ruby Matilda’ showed marked increase in the cytokinin precursors and derivatives including adenosine, and its deaminated form, inosine in trees with high return bloom. The compounds showed significant correlation with return bloom, both as local signalling and as a whole tree response. Inosine is a purine nucleoside that is a constituent of DNA and RNA and a precursor of cytokinin, a chemically diverse class of plant hormones associated with a range of actions on plant growth and development. Application of inosine to seeds of rice, tomato, onion, sunflower and soybean grown in a hydroponic system resulted in improved growth of all plant parts, particularly root growth, with the exception of soybean [25]. In the present study, the pyrimidine metabolite, ureidopropionic acid, an intermediate in uracil metabolism, also increased in high-return-bloom trees. Proteomic studies on early flower bud development mechanism in OFF apple trees indicated the involvement of purine and pyrimidine metabolism, as well as flavonoid biosynthesis [1].

The increased levels of compounds identified in the FI phase of buds collected from ‘Ruby Matilda’ trees with high return bloom could be candidates for improving return bloom and mitigating biennial bearing via exogenous application. Current thinning agents utilised by the industry are thought to potentially induce return bloom when fruitlets are removed prior to FI or at the early stages of fruitlet development of 8–15 mm in size. For example, the synthetic derivative of cytokinin BAP (thinning agent), is known to promote return bloom when application is performed during the critical FI period. Similarly, the natural phytohormone abscisic acid has been known to be an effective thinning agent with some cultivars and increases return bloom only in combination with BA. However, in this study, the cytokinin derivatives inosine and its bound derivatives, as well as ABA, were found to be significantly involved in FI. Based on previous studies, it was unclear whether cytokinins and ABA thinning agents were flower-promoting or abscission-related metabolites [26]. However, this study provided evidence that cytokinins and ABA are directly involved in FI; thus, the mechanism of thinning is likely the activation of a hormone signal transduction pathway, which likely leads to abscission of fruit. Leaves are known as promoters of FI and are also a major source of cytokinins. Metabolic profiling of leaves could improve the understanding of sink–source relationships in FI.

While exogenous application of cytokinins has been linked to improve rate of floral stimuli and bloom in apple, endogenous levels of cytokinin-related metabolites in the bud meristem have only been previously identified in the form of an adenine derivative [1]. Corbesier et al. [27] described the role of cytokinins during floral transition of the long-day plant *Arabidopsis thaliana* (L.) Heynh. The study describes that in response to a 16 h photoperiod, the leaf tissues and leaf phloem exudate contained increased levels of isopentenyl adenine, and at 20 h, the shoot apical meristem increased in both isopentenyladenine and zeatin in induced plants when compared to vegetative controls. Similarly, in strawberry, free cytokinins were significantly higher in shoot tips when compared to controls [28]. There are also examples where exogenous application of rare earth metal nitrates such as lanthanum and cerium and/or long-chain primary alcohol triacontanol manipulates the endogenous cytokinins for early flowering in *A. thaliana* [29,30] and boosts quality and yield of flowers in other plants such as orchids and *Chrysanthemum* × *morifolium* (Ramat.) Hemsl. Although the mechanism is unclear, the potential to improve endogenous cytokinin concentrations without the use of hormonal agents could be useful for the apple industry.

The flavonoids quercetin, epicatechin, naringenin, eriodictyol, avicularin and sinensin showed varied levels with high and low treatments. Kaempferol and its precursor naringenin are important biomarkers, as previous reports have identified derivatives of its biosynthetic intermediate, p-coumaryl COA in ‘ON’ trees [1]. Moreover, the transcriptome and proteome of apple buds showed enzymatic activity of enzymatic reaction EC:2.3.1.133 that metabolizes at least three derivatives of p-coumaric acid, including p-coumaroyl CoA and caffeoyl shikimic acid, precursors of the kaempferol and chlorogenic acid pathway [1]. Nuclear localisation of flavonoids has been reported in many plant species, suggesting that flavonoids may function in transcriptional regulation of endogenous gene expression [31]. Naringenin chalcone and apigenin may also influence flavonoid biosynthesis by regulating transcription of flavonoid biosynthetic enzymes [32]. The increased levels of the phenylpropanoid pathway intermediates, including hydroxy- and methoxy-cinnamates observed in the present study, are consistent with investigations related to the strongly biennial cultivar ‘Nicoter’ [21], where increased levels of cinnamates and salicylate derivatives indicated involvement of the phenylalanine ammonia lyase pathway (PAL) pathway.

The amino acids leucine and L-carnitine and the peptide aspartyl-aspartate were observed in high-return-bloom trees. Amino acids serve as protein building blocks and increased levels could suggest resources/nutrients for floral organ development.

Using ESI LCMS profiling, and MSMS fragmentation techniques, many of the differentially expressed metabolites in ‘Ruby Matilda’ cultivars were identified. Although further annotation of the “unknown” metabolites would be required to characterise the full extent of the metabolic pathways driving biennial bearing, most of the classes of compounds and derivatives identified in this study and previous studies give confidence on the involvement of the phenylpropanoid and cytokinin pathway in FI, as described in the KEGG plant hormone biosynthesis map [33] (Figure S3). Moreover, the compounds identified corroborate previous investigations on FI pathways in apple cultivars [1,21].

## 5. Conclusions

This study showed that the unique metabolite expression in high-return-bloom ‘Ruby Matilda’ trees is likely associated with FI. The increased levels of ABA, a compound typically associated with fruit abscission, is a notable response and further advances our understanding of the interactions of FI and carboyhydrate levels in trees. Moreover, the diverse and distinct compounds identified in the cytokinin pathway, including the inosines and adenosine derivatives as well as the phenylpropanoid pathway, further support their involvement in FI. Further validation studies would be required to confirm the source of the differentially expressed metabolites as well as their function in other plant tissues. Together these studies provide insight into bud metabolite expression in response to varying levels of flower numbers in the tree as well as within its leaders/branches, representing a zonal signal response. Findings obtained on the cultivar ‘Ruby Matilda’ are relevant for other ‘Cripps Pink’ sports marketed as Pink Lady^®^, that together account for 41% of the Australian apple production [34].

## Figures and Tables

**Table 1:** Negative mode LC-MSMS data that were significant (VIP > 2.0) in the OPLSDA model (RT^HIGH^ vs RT^LOW^) with associated effect size. *P* values indicate significance of linear model (y (metabolite response) ~ flower clusters) in ‘Ruby Matilda’.

Identity	Compound Class	Retention Time (min)	Mass (*m*/*z*) [M − H]−	Molecular Formula	Mass Error (ppm)	VIP Score	Effect Size	*	*p*-Value	MS^2^ Ions	Metabolite Level
coumaric acid derivative	phenylpropanoid	3.85	429.1407	C_19_H_26_O_11_	−3.7	2.78	2.36	↑	6.7 × 10^−7^	361.1503, 265.0700, 163.0391, 145.0281, 117.0335, 59.0126	3
coumaric acid derivative	phenylpropanoid	3.78	459.1512	C_20_H_28_O_12_	−3.3	2.73	2.18	↑	8.9 × 10^−5^	265.0715, 235.0715, 205.0500, 163.0391, 145.0285, 119.0492, 117.0335, 89.0233, 59.0126	3
sinapaldehyde	phenylpropanoid	4.29	369.1195	C_17_H_22_O_9_	−4.1	2.51	1.88	↑	5.7 × 10^−2^	359.1194, 163.0392, 145.0295, 117.0336	2
coumaric acid derivative	phenylpropanoid	5.57	473.1452	C_24_H_26_O_10_	−2.1	2.37	1.47	↑	2.7 × 10^−3^	307.0826, 273.0771, 165.0549, 150.0313, 145.0285, 123.0441, 117.0336	2
coumaric acid derivative	phenylpropanoid	4.57	383.1352	C_18_H_24_O_9_	−3.9	2.36	1.92	↑	1.7 × 10^−1^	383.1356, 163.0391, 145.0286, 117.0335	2
		2.20	206.9884			2.35	1.44	↓	1.7 × 10^−1^		
coumaric acid derivative	phenylpropanoid	5.38	487.1246	C_24_H_24_O_11_	−2.3	2.35	1.40	↑	5.5 × 10^−4^	341.0885, 179.0345, 161.0239, 145.0288, 135.0444, 117.0338	2
coumarate diglucoside	phenylpropanoid	3.70	489.1618	C_21_H_30_O_13_	−3.1	2.21	2.07	↑	2.5 × 10^−4^	163.0393, 145.0286, 119.0492	2
		5.34	503.1557	−		2.18	1.31	↑	1.3 × 10^−1^	−	5
		3.09	427.0859	−		2.16	1.49	↑	1.0 × 10^−1^	−	5
		3.09	359.0990	−		2.15	1.54	↑	1.5 × 10^−1^	−	5
		1.32	209.0661	−		2.13	1.55	↑	6.0 × 10^−3^	−	5
quercetin 3-(6″-malonyl-glucoside)	flavanol	4.79	549.0890	C_24_H_22_O_15_	−2.7	2.09	1.85	↓	7.3 × 10^−5^	As per + ve mode (Table 2)	3
		4.85	433.0469	−		2.07	1.48	↓	3.5 x10^−5^	−	5
		4.42	307.1400	−		2.05	1.38	↑	2.1 x10^−2^	−	5
		5.06	475.1250	−		2.03	1.69	↑	1.1 × 10^−1^	−	5
		4.82	501.0649	−		2.01	1.15	↓	8.9 × 10^−5^	−	5
abscisate glucoheptonic acid	abscisic acid	4.81	471.1869	C_22_H_32_O_11_	−1.7	2.43	1.52	↑	3.8 x10^−3^	263.1291, 219.1391, 151.0755, 125.0600	2
inosine derivative	cytokinin	3.89	439.1827	C_19_H_28_N_4_O_8_	−0.9	2.42	1.22	↑	1.8 × 10^−3^	393.1770, 325.1147, 265.0931, 205.0712, 163.0604	3
inosine diglucoside	cytokinin	1.23	475.1781	C_29_H_24_O_3_N_4_	−3.4	2.18	2.62	↑	3.3 × 10^−3^	343.1250, 179.0551, 131.0452, 113.0346	2
inosine derivative	cytokinin	1.26	473.1625	C_19_H_28_N_3_O_11_	3.2	2.01	2.16	↑	8.5 × 10^−3^	341.1106, 179.0556, 131.0453, 113.0347	3
inosine	cytokinin	1.27	267.0723	C_10_H_12_N_4_O_5_	0.4	2.20	1.31	↑	2.8 × 10^−3^	249.0614, 207.0509, 191.0558, 175.0245, 113.0234, 85.0284	2
ureidopropionic acid	cytokinin	1.18	131.0452	C_4_H_8_N_2_O_3_	−0.8	2.00	2.10	↑	5.7 × 10^−2^	114.0188, 113.0348, 95.0241	2

* ↑ = up-regulated in RT^HIGH^, ↓ = down-regulated in RT^HIGH^.

**Table 2:** Positive mode LC-MSMS data that were significant (VIP > 2.0) in the OPLSDA model (RT^HIGH^ vs RT^LOW^) with associated effect size. *P* values indicate significance of linear model (y (metabolite response) ~ flower clusters) in ‘Ruby Matilda’.

Identity	Compound Class	Retention Time (min)	Mass (*m*/*z*) [M + H]+	Molecular Formula	Mass Error (ppm)	VIP Score	Effect Size	*	*p*-Values	MS^2^ Ions	Metabolite Level
epicatechin-3′-O-glucuronide	flavanol	3.85	453.1365	C_21_H_24_O_11_	5.7	2.53	2.23	↑	3.7 × 10^−6^	291.0855, 147.0446, 139.0389, 123.0443	2
cytokinin derivative	cytokinin	4.81	449.1775	C_16_H_26_N_5_O_10_	−5.1	2.46	1.53	↑	1.4 × 10^−1^	287.1249, 269.1140, 185.0420, 153.0185	2
inosine diglucoside	cytokinin	1.23	477.1919	C_29_H_24_N_4_O_3_	−0.4	2.39	3.14	↑	4.9 × 10^−3^	133.0608, 116.0344, 87.0556, 74.0241	2
inosine derivative	cytokinin	1.28	475.1765	−	−	2.39	3.07	↑	2.0 × 10^−1^	133.0610, 116.0346, 87.0557, 74.0241	3
		4.82	267.0431	−	−	2.31	1.14	↓	2.5 × 10^−1^		5
		5.57	497.1410	−	−	2.28	1.61	↑	2.4 × 10^−3^	-	
quercetin 3-(6″-malonyl-glucoside)	flavanol	4.79	551.1024	C_24_H_22_O_15_	1.3	2.26	1.90	↓	6.7 × 10^−3^	303.0497, 257.0431, 231.0494, 153.0187	2
(2R)-3-(3,4-dihydroxyphenyl)-2-hydroxypropanoate	phenylpropanoid	3.09	199.0601	C_9_H_10_O_5_	0.0	2.25	1.49	↑	6.0 × 10^−2^	155.0702, 140.0468, 123.0442, 95.0495	2
	flavanol	4.83	454.0647	−	−	2.25	1.27	↓	5.3 × 10^−3^	−	5
		1.21	315.1392	−	−	2.25	2.34	↑	7.1 × 10^−2^	−	5
		4.57	407.1309	−	−	2.25	1.82	↑	7.8 × 10^−3^	−	2
		4.82	671.1072	−	−	2.20	1.66	↓	5.8 × 10^−3^	−	5
		9.61	537.3027	−	−	2.19	1.78	↑	9.6 × 10^−4^	−	5
		1.82	256.1288	−	−	2.19	2.16	↑	1.7 × 10^−2^	−	5
ureidopropionic acid	cytokinin	1.19	133.0606	C_4_H_7_N_2_O_3_		2.18	2.70	↑	6.1 × 10^−2^	116.0344, 87.0557, 74.0241	2
		1.34	256.1287	−		2.16	2.12	↑	3.1 × 10^−2^	−	5
		1.28	112.9999	−		2.15	1.68	↑	1.7 × 10^−3^	−	5
		3.09	383.0949	−		2.15	1.42	↑	7.1 × 10^−2^	−	5
leucine	amino acid	2.40	132.1020	C_6_H_13_NO_2_	−0.8	2.11	1.57	↑	6.3 × 10^−2^	86.0968, 69.0703	2
		1.27	85.0287	C_4_H_4_O_2_	−3.5	2.11	1.18	↓	9.3 × 10^−5^	−	
L-carnitine	amino acid	1.25	162.1122			2.04	2.51	↑	9.5 × 10^−2^	144.0656, 116.0709, 98.0604	2
aspartyl-aspartate	dipeptide	1.32	249.0713	C_8_H_12_N_2_O_7_	1.7	2.02	2.62	↑	9.4 × 10^−3^	232.0450, 214.0347, 204.0502, 186.0398	2
aminoisobutyrate	cytokinin	1.22	104.0708	C_4_H_9_NO_2_	−1.9	2.02	1.78	↑	4.4 × 10^−2^	87.0445, 60.0812, 58.0655	2
		4.81	891.1567	−	−	2.02	1.29	↓	1.9 × 10^−3^	−	
methoxy coumarin	phenylpropanoid	5.84	177.0546	C_10_H_8_O_3_	0.0	2.00	1.27	↑	1.7 × 10^−2^	145.0283, 117.0335, 89.0388	2

* ↑ = up-regulated in RT^HIGH^, ↓ = down-regulated in RT^HIGH^.

**Table 3:** Negative mode LC-MSMS data that were significant (VIP > 2.0) in the OPLSDA model (RL^HIGH^ vs RL^LOW^) with associated effect size. *P* values indicate significance of linear model (y (metabolite response) ~ flower clusters) in ‘Ruby Matilda’.

Identity	Compound Class	Retention Time (min)	Mass (*m*/*z*) [M − H]−	Molecular Formula	Mass Error (ppm)	VIP Score	Effect Size	*	*p*-Value	MS^2^ Ions	Metabolite Level
quercetin-3-arabinoside	flavanol	4.87	433.0775	C_20_H_18_O_11_	−2.3	2.3	1.2	↓	3.1 × 10^−2^	300.0279, 271.0252, 255.0298, 243.0299	2
naringenin glucoside	flavanol	5.42	433.1147	C_21_H_22_O_10_	4.1	2.2	1.5	↓	1.1 × 10^−2^	271.0602, 255.0299, 253.0505, 151.0029,	2
coumaric acid	phenylpropanoid	3.70	489.1618	C_21_H_30_O_13_	−3.1	2.2	1.6	↑	3.9 × 10^−4^	325.1147, 265.0719, 163.0393, 145.0286,	2
gingerol	phenolic	8.70	293.1762	C_17_H_26_O_4_	−5.1	2.1	1.1	↓	2.1 × 10^−1^	249.1861, 193.1593, 136.0885	2
sinensin	flavanol	5.17	449.1094	C_21_H_22_O_11_	−3.6	2.1	3.2	↓	1.0 × 10^−2^	287.0566, 151.0028, 135.0442, 107.0128	2
apigenin-O-(malonyl-glucoside)	flavanol	5.17	517.0967	C_24_H_22_O_13_	1.9	2.1	3.5	↓	3.6 × 10^−1^	-	3
dalpatein-apiofuranosyl-glucopyranoside	flavanol	4.96	635.1596	C_29_H_32_O_16_	1.7	2.1	1.6	↓	1.8 × 10^−2^	589.1551, 567.1729, 463.0878, 316.0229	2
tetrahydroxyanthraquinone	quinone	3.70	375.0698	C_18_H_16_O_9_	−3.5	2.1	1.4	↓	1.9 × 10^−4^	357.0582, 201.0164, 189.0163, 179.0342, 161.0235, 135.0442	2
hydroxycinnamate	phenylpropanoid	5.57	473.1452	C_24_H_26_O_10_	−2.1	2.1	1.4	↑	1.5 × 10^−2^	307.0826, 273.0771, 165.0549, 150.0313, 145.0285, 123.0441,117.0336	2
coumarate glycoside	phenylpropanoid	3.78	459.1512	C_20_H_28_O_12_	−3.3	2.0	1.6	↑	8.5 × 10^−4^	265.0715, 235.0715, 205.0500, 163.0391, 145.0285, 119.0492, 117.0335, 89.0233, 59.0126	2
methoxy cinnamate	phenylpropanoid	6.44	177.0550	C_10_H_10_O_3_	−2.3	2.0	1.8	↑	4.0 × 10^−6^	162.0315, 145.0267, 123.0442, 121.0286	2
	phenylpropanoid	4.94	567.1719			2.0	1.5	↑	4.6 × 10^−2^	273.0772, 167.0343, 123.0441, 81.0334	4
hydroxycinnamate	phenylpropanoid	3.85	429.1407	C_19_H_26_O_11_	−3.7	2.0	1.6	↑	2.8 × 10^−4^	361.1503, 265.0700, 163.0391, 145.0281, 117.0335	2
	-	4.78	737.1725			2.0	2.8	↓	6.7 × 10^−6^	575.1197, 407.0775, 395.0775, 243.0297	5

* ↑ = up-regulated in RL^HIGH^, ↓ = down-regulated in RL^HIGH^.

**Table 4:** Positive mode LC-MSMS data that were significant (VIP > 2.0) in the OPLSDA model (RL^HIGH^ vs RL^LOW^) with associated effect size. *P* values indicate significance of linear model (y (metabolite response) ~ flower clusters) in ‘Ruby Matilda’.

Identity	Compound Class	Retention Time (min)	Mass (*m*/*z*) [M + H]+	Molecular Formula	Mass Error (ppm)	VIP Score	Effect Size	*	*p*-Value	MS^2^ Ions	Metabolite Level
naringenin	flavanol	3.41	273.0756	C_15_H_12_O_5_	0.4	2.56	2.14	↑	1.5 × 10^−5^	243.0650, 215.0702, 151.0389, 123.0442	2
		1.44	247.0422	−	−	2.47	1.58	↑	1.3 × 10^−4^	229.0319, 173.0209, 97.0283	5
		1.38	261.0577	−	−	2.44	1.57	↑	5.5 × 10^−4^	−	5
		1.42	324.0341	−	−	2.42	1.75	↑	2.6 × 10^−3^	−	5
		4.57	407.1309	−	−	2.34	1.46	↑	2.2 × 10^−3^	−	5
		1.24	295.0784	−	−	2.25	1.69	↑	9.0 × 10^−4^	−	5
flavanol derivative	flavanol	5.42	435.1279	C_21_H_22_O_10_	1.6	2.21	1.62	↓	1.4 × 10^−2^	303.0498, 273.0754, 229.0493, 153.0181	3
		4.81	891.1567	−	−	2.18	1.35	↓	1.9 × 10^−2^	−	
hydroxycinnamic acid	phenylpropanoid	3.80	165.0544	C_9_H_8_O_3_	1.2	2.15	1.57	↑	5.7 × 10^−3^	147.0441, 119.0493, 91.0546	2
		1.28	112.9999	−	−	2.14	1.67	↑	1.1 × 10^−3^	−	5
		5.17	451.1233	−	−	2.11	2.03	↓	9.1 × 10^−3^	−	5
		5.42	273.0756	−	−	2.11	1.55	↓	2.0 × 10^−2^	−	5
avicularin (flavanol glucoside)	flavanol	4.89	435.0913	C_20_H_18_O_11_	2.1	2.09	1.28	↓	9.7 × 10^−2^	303.0498, 273.0757, 229.0494, 153.0182	2
adenosine	cytokinin	1.32	268.1035	C_10_H_13_N_5_O_4_	1.9	2.08	2.60	↑	2.1 × 10^−2^	245.2293, 136.0617, 91.0577, 77.0421	2
unknown cytokinin derivative glucoside	cytokinin	5.57	497.1410	C_19_H_22_N_5_O_11_	−4.2	2.06	1.44	↑	1.8 × 10^−3^	331.0780, 189.0524	3
eriodictyol	flavanol	5.17	289.0703	C_15_H_12_O_6_	1.4	2.04	4.10	↓	2.2 × 10^−2^	163.0390, 153.0182, 135.0441, 123.0442	2
		5.74	277.0640	−	−	2.01	1.40	↑	1.9 × 10^−3^	−	5
adenosine 5′-monophosphate	cytokinin	1.32	348.0699	−	−	2.00	1.62	↑	3.1 × 10^−3^	−	2
		3.69	491.1752	−	−	2.00	1.47	↑	5.5 × 10^−4^	−	

* ↑ = up-regulated in RL^HIGH^, ↓ = down-regulated in RL^HIGH^.

## Data Availability

The datasets generated during the current study are available from the corresponding author upon reasonable request and are not publicly available due to commercial restrictions.

## Supplementary Materials

The following supporting information can be downloaded at: https://www.mdpi.com/article/10.3390/metabo14050251/s1. Figure S1: PCA scores plot of (**a**) ESI+ UHPLC-HRMS and (**b**) ESI− UHPLC-HRMS data acquired from the aqueous extracts of apple spur buds of ‘Ruby Matilda’. Figure S2: PCA scores plot of ESI+ UHPLC-HRMS of (**a**) RL^HIGH^ vs RL^LOW^ (**b**) RT^HIGH^ vs RT^LOW^ and ESI− UHPLC-HRMS of (**c**) RL^HIGH^ vs RL^LOW^ (**d**) RT^HIGH^ vs RT^LOW^, acquired from the aqueous extracts of apple spur buds of ‘Ruby Matilda’ crop load treatments: RT^HIGH^ (12.6–20.0 flower no. cm^−2^ leader cross-sectional area (LCSA); 4.0–10.1 fruit no. cm^−2^ LCSA; *n* = 12), RL^HIGH^ (15.4–21.6 flower no. cm^−2^ LCSA of leader; 3.3–17.2 fruit no. cm^−2^ LCSA of leader; *n* = 12), RT^LOW^ (2.93–4.48 flower no. cm^−2^ LCSA; 2.24–6.46 fruit no. cm^−2^ LCSA; *n* = 12), and RL^LOW^ (2.09–3.95 flower/cm^2^ LCSA of leader; 5.0–7.5 fruit no. cm^−2^ LCSA of leader; *n* = 12). Figure S3: Plant hormone biosynthetic map sourced from KEGG pathways identifying key plant hormones (auxins, cytokinins, abscisates and salicylates) and compounds reported in the present study and previous literature that are associated with floral induction in apple.
