# Supplementary material for: Zonal Chemical Signal Pathways Mediating Floral Induction in Apple
Source: Metabolites. 2024 Apr 25;14(5):251. doi: 10.3390/metabo14050251 (PMC11123431; doi:10.3390/metabo14050251)
Supplement: Supplementary file 1 [file metabolites-14-00251-s001.zip › metabolites-2848764-supplementary.pdf]

metabolites

Article

Zonal Chemical Signal Pathways Mediating Floral Induction in Apple

Priyanka Reddy [1,2,*], Tim Plozza [1], Alessio Scalisi [3], Vilnis Ezernieks [1], Ian Goodwin [3,4] and Simone Rochfort [1,2,*]

1 Agriculture Victoria, AgriBio, Centre for AgriBioscience, Bundoora, VIC 3083, Australia
2 School of Applied Systems Biology, La Trobe University, Bundoora, VIC 3083, Australia
3 Tatura SmartFarm, Agriculture Victoria, Tatura, VIC 3616, Australia
4 Centre for Agricultural Innovation, University of Melbourne, Parkville, VIC 3010, Australia
* Correspondence: priyanka.reddy@agriculture.vic.gov.au (P.R.); simone.rochfort@agriculture.vic.gov.au (S.R.)

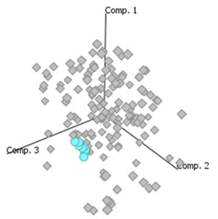

(**a**) ESI+ UHPLC-HRMS

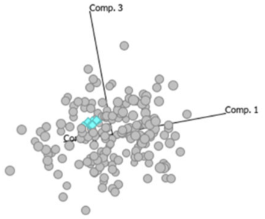

(**b**) ESI- UHPLC-HRMS

Figure S1. PCA scores plot of (**a**) ESI+ UHPLC-HRMS and (**b**) ESI- UHPLC-HRMS data acquired from the aqueous extracts of apple spur buds of 'Ruby Matilda'.

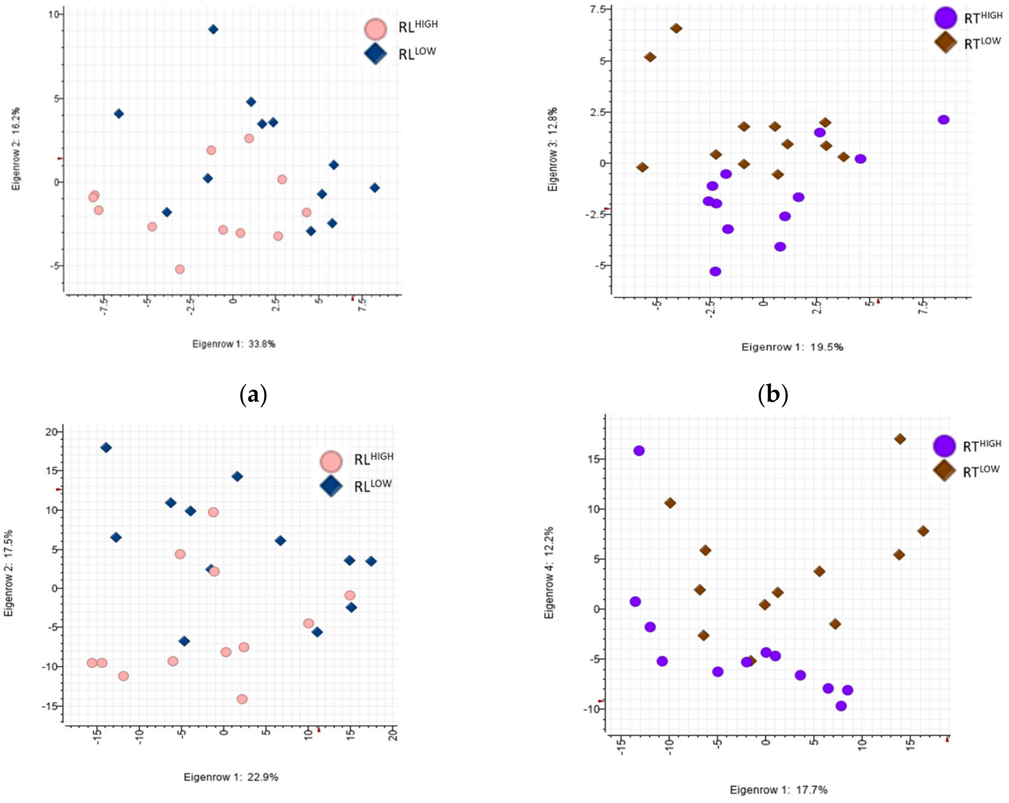

(**a**) (**b**)

(**c**) (**d**)

Figure S2. PCA scores plot of ESI+ UHPLC-HRMS of (**a**) RL^{HIGH} vs RL^{LOW} (**b**) RT^{HIGH} vs RT^{LOW} and ESI− UHPLC-HRMS of (**c**) RL^{HIGH} vs RL^{LOW} (**d**) RT^{HIGH} vs RT^{LOW} , acquired from the aqueous extracts of apple spur buds of 'Ruby Matilda' crop load treatments: RT^{HIGH} (12.6–20.0 flower no. cm^{-2} leader cross-sectional area (LCSA); 4.0–10.1 fruit no. cm^{-2} LCSA; n = 12), RL^{HIGH} (15.4–21.6 flower no. cm^{-2} LCSA of leader; 3.3–17.2 fruit no. cm^{-2} LCSA of leader; n = 12), RT^{LOW} (2.93–4.48 flower no. cm^{-2} LCSA; 2.24–6.46 fruit no. cm^{-2} LCSA; n = 12), and RL^{LOW} (2.09–3.95 flower/cm^2 LCSA of leader; 5.0–7.5 fruit no. cm^{-2} LCSA of leader; n = 12).

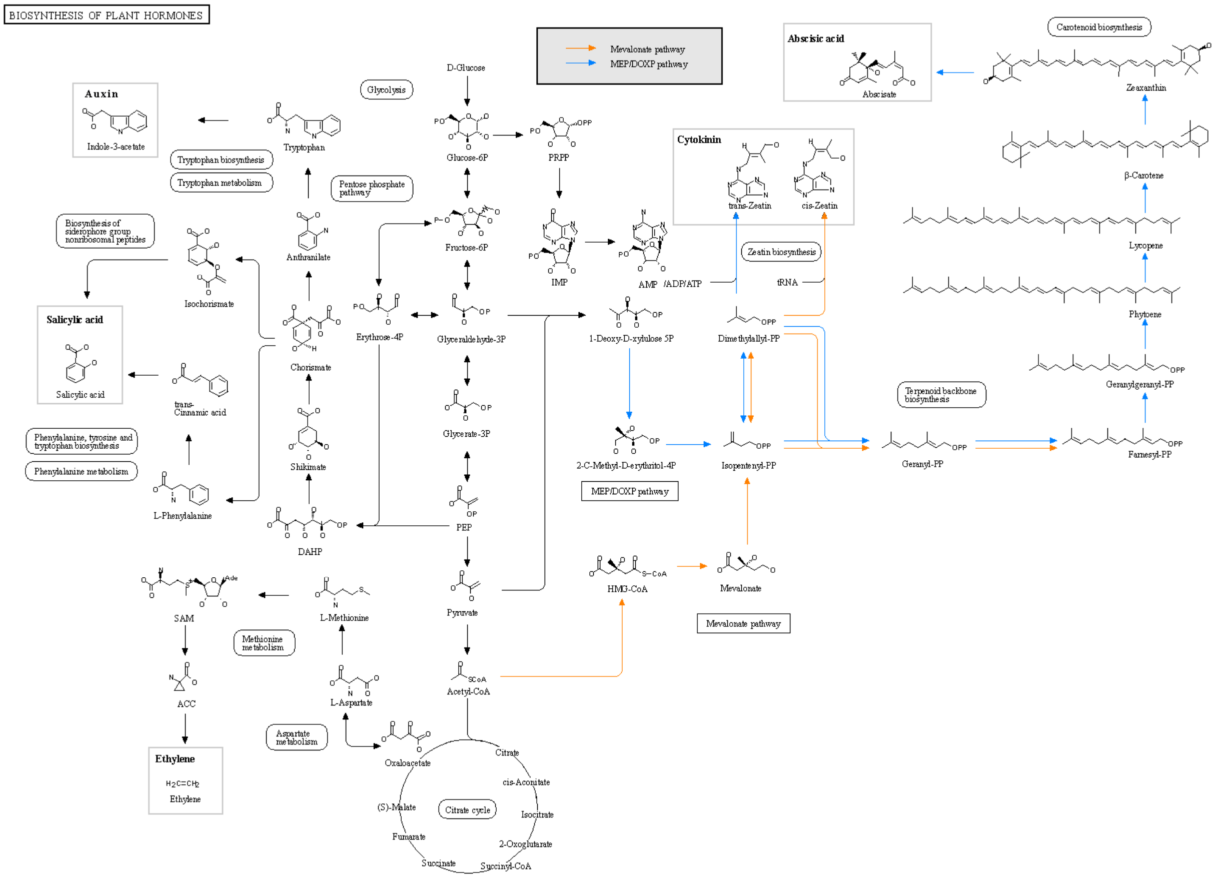

Figure S3. Plant hormone biosynthetic map sourced from KEGG pathways identifying key plant hormones (auxins, cytokinins, abscisates and salicylates) and compounds reported in the present study and previous literature that are associated with floral induction in apple.
